# Supplementary material for: Content-rich biological network constructed by mining PubMed abstracts
Source: BMC Bioinformatics. 2004 Oct 8;5:147. doi: 10.1186/1471-2105-5-147 (PMC528731; doi:10.1186/1471-2105-5-147)
Supplement: Additional File 2 — The original results of the above study (non-essential files are deleted to keep the file size under the limit set by BMC bioinformatics). [file 1471-2105-5-147-S2.bz2 › chilibotAdditionalFile2/dip05/694ID11994151E18019/html/TOLA_YBGF.html]

 


 **TOLA** and **YBGF** 
  
Found 1 abstracts in PubMed, retrieved 1.  
 

 What does Google say? 
 PDF only 
| .edu only 

---

**Interactive relationship** (e.g. stimulation, inhibition, etc)

**Neutral relationship**- TolB dimerizes and the periplasmic domain of  **TolA**  interacts with  **YbgF**  and TolB.  Ref: 11994151 Mol Microbiol, 2002
- Our results indicate that the central domain of  **TolA**  TolAII is sufficient to interact with  **YbgF** , that the C terminal domain of  **TolA**  TolAIII is sufficient to interact with TolB,.  Ref: 11994151 Mol Microbiol, 2002

**Non-interactive relationship** (e.g. studied together, co-existance, homology, etc.)

- The Tol Pal system of Escherichia coli is composed of the YbgC, TolQ,  **TolA** , TolR, TolB, Pal and  **YbgF**  proteins.  Ref: 11994151 Mol Microbiol, 2002
